# Transdermal laser for facial vascular lesions: a single center experience

**DOI:** 10.1590/1677-5449.202301032

**Published:** 2025-10-27

**Authors:** Felipe Coelho, Fernando Tres Silveira, Paulo Roberto Bignardi, Sergio Quilici Belczak, Walter Junior Boim de Araujo, Rodrigo Gomes de Oliveira

**Affiliations:** 1 Pontificia Universidade Catolica do Parana – PUCPR, Londrina, PR, Brasil.; 2 Cardiodiagnóstico, Vitória, ES, Brasil.; 3 Centro Universitário São Camilo – CUSC, São Paulo, SP, Brasil.; 4 Universidade Federal do Paraná – UFPR, Curitiba, PR, Brasil.

**Keywords:** laser therapy, vascular, telangiectasia, laser, laserterapia, vascular, telangiectasia, laser

## Abstract

**Background:**

Facial vascular lesions are a common situation in medical practice and can cause cosmetic concerns for patients.

**Objectives:**

To evaluate the results of facial telangiectasia treatment using the Nd:YAG 1064nm transdermal laser.

**Methods:**

A retrospective study was conducted in which two independent evaluators evaluated pre- and post-treatment photographic records of patients submitted to Nd:YAG 1064nm transdermal laser treatment for facial vascular lesions.

**Results:**

Both evaluators’ ratings indicated a statistically significant improvement after treatment (p<0.0001), with a moderate strength of agreement between evaluators.

**Conclusions:**

Limitations notwithstanding, the results suggest that the use of 1064 nm Nd:YAG transdermal laser for the treatment of facial vascular lesions proved to be effective for treating facial vascular lesions.

## Resumo

**Contexto:**

Lesões vasculares faciais são uma situação comum na prática médica e podem gerar preocupações estéticas para os pacientes.

**Objetivos:**

Avaliar os resultados do tratamento de telangiectasias faciais com *laser* transdérmico Nd:YAG 1064 nm.

**Métodos:**

Foi realizado um estudo retrospectivo de pacientes submetidos a tratamento transdérmico com *laser* Nd:YAG 1064 nm para lesões vasculares faciais. Dois avaliadores independentes analisaram registros fotográficos obtidos antes e após o tratamento.

**Resultados:**

Ambos os avaliadores relataram melhora estatisticamente significativa após o tratamento (p < 0,0001), com concordância de força moderada entre eles.

**Conclusões:**

Apesar das limitações, os resultados sugerem que o uso de *laser* transdérmico Nd:YAG 1064 nm é eficaz no tratamento de lesões vasculares da face.

## INTRODUCTION

Located on the nose and lateral cheeks, facial telangiectasia can be defined as superficial cutaneous vessels visible to human eye.^1^ Cosmetic concerns often require a range of different treatment options.

In contrast, reticular veins are vessels located in the subcutaneous tissues, are blue in color, measure between 1 and 3mm, and trace a tortuous path in the periorbital area.

Aging is the most common factor associated with development of facial telangiectasia and reticular veins. Depending on coloration, telangiectasias can be associated with arterial origin when small in diameter, bright red, and with no protrusion beyond the surface of the skin. Characteristic indicative of venous origin include larger width, blue color, and, frequently, protrusion beyond the skin.^2^

Besides aging, facial telangiectasias have other causes, such as: rosacea, topical steroids, hyperestrogenism, liver disease, connective tissue diseases, and scarring, among others.^3^

Given the fact that cosmetic complaints comprise almost the entirety of treatment demand, therapeutic options must be risk-free and should not cause unsightly scarring.^1^

Therapeutic options for treating facial telangiectasia and reticular veins include electrocoagulation, radiofrequency, sclerotherapy, microsurgery, and laser therapy. When well executed, these techniques offer good results for clearing telangiectasias and periorbital reticular veins.^3^

However, laser therapy offers theoretical advantages when compared with sclerotherapy, including minimizing telangiectasic matting, hyperpigmentation associated with hemosiderin deposition, and potential allergenic effects.^4^

Moreover, laser therapy avoids needles and more invasive treatments.

Continuous-wave argon and carbon dioxide lasers have previously been employed for telangiectasia treatment, with good results.^5^

Alternatively, long pulse devices such as Nd:YAG 1064nm lasers allow deeper delivery of energy. Using a small spot size, energy can be focused on superficial areas, allowing treatment of telangiectasias.

From this perspective, both superficial and deep vessels can be treated with this technology.

This study aimed to evaluate the results of implementation of long pulse Nd:YAG 1064nm laser as a routine practice for treatment of patients with vascular facial lesions.

## METHODS

A retrospective study was conducted to evaluate information obtained from the medical records of patients treated at a single hospital from October 2021 to May 2022.

The following outcomes were evaluated:

- Lesion whitening;
- Complications;
- Patient satisfaction.

The study selected photographic records of consecutive patients aged over 18 years presenting with vascular lesions on the face, such as telangiectasias, periorbital reticular veins, and venous malformations involving the lips, who were treated with long pulse Nd: YAG 1064nm laser.

Photographic records of patients before and after treatment were analyzed by 2 independent physicians to evaluate treatment outcomes. The evaluators were asked to assign scores from 0 to 5 for the improvement observed between the before and after photographic records.

Side effects such as skin burns, skin retraction, pigmentation, phlebitis, mucosa retraction, and bleeding were assessed to evaluate complication outcomes.

Patients’ satisfaction was rated at the end of treatment by the treating physician, evaluating how satisfied each patient was with their treatment: fully satisfied, partially satisfied, or unsatisfied.

All treatments were performed by 2 vascular surgeons using the same long pulse Nd: YAG 1064nm laser device (Etherea MX – Vydence Medical – São Carlos, SP).

Medical records were excluded if data were missing such as patients’ initial and final photographic records or the laser parameters employed, as were the medical records of patients who had been treated by a different vascular surgeon.

The following variables were assessed: vascular lesion type (telangiectasia, reticular vein, or venous malformation), vascular lesion location on the face, number of sessions, laser parameters employed, lesion whitening scored by external evaluators, and patient satisfaction.

Informed consent for laser treatment was obtained from each patient, who also granted permission for use of their clinical data for scientific investigations. The present study was conducted with the approval of the local ethics committee (Number 46601321.0.0000.0099 and substantiated opinion number 5.049.484) and followed the STROBE Initiative guidelines. All procedures were in accordance with the ethical standards of the 1964 Helsinki declaration and its later amendments or comparable ethical standards.

### Transdermal laser technique

The selected medical records were for patients who had their procedures performed at the outpatient clinic of the Hospital Vascular de Londrina (Brazil).

All patients were evaluated by clinical consultation and physical examination. Examinations with the augmented reality device VeinViewer (Christie Medical - Cypress, CA) was used to identify the periorbital reticular veins, or feeder veins related to facial telangiectasias.

All treatments were performed by 2 certified vascular surgeons using the same long pulse Nd: YAG 1064nm laser device (Etherea MX – Vydence Medical – São Carlos, SP).

Facial telangiectasias were treated using 2 and 3mm spot sizes, 10-20ms pulse duration, and fluence ranging from 150 to 200J/cm^2^. Periorbital reticular veins were treated using the 6mm spot size, 20-40ms pulse duration, and fluence ranging from 50 to 70J/cm^2^. Labial venous malformations were treated using the 6mm spot size, 30-50ms pulse duration, and fluence ranging from 40 to 60J/cm^2^.

The spot size employed determines the distance between laser shots: for the 2-3mm spot size the distance was 2mm, and the distance for the 6mm spot size was 5mm. Stacking was avoided at all costs.

All procedures were executed under a skin cooling device (Siberian Fit – Vydence Medical – São Carlos, SP) and all procedures were executed while wearing protective glasses.

All patients were discharged after the procedure with instructions to avoid sun exposure for 3-5 days and encouraged to perform skin moisturization, with no other restrictions.

A 30-day follow-up consultation was scheduled to assess the need for additional sessions.

Patients with incomplete results were treated again using the same procedure.

The safety endpoints were no skin burns, skin retraction, phlebitis, bleeding, pigmentation, or mucosa ulceration.

### Statistical analysis

Given that the standard deviation or population frequencies of the variable under study are unknown and there are no similar data available in the literature, a pre-test was carried out with 30-40 individuals and the behavior of this subgroup was considered for the population estimate.

Data were analyzed using MedCalc for Windows, version 9.5.2.0 (MedCalc Software, Mariakerke, Belgium). We used the *t* test for independent samples for continuous variables with normal distribution, the Mann-Whitney U test for continuous variables with skewed distribution, and the chi-square test for categorical variables. We used the Welch test for unequal variances. We set the level of significance at 5% and calculated the power as (1 – β) = 1.0 for all tests. Categorical data were expressed as frequencies and continuous variables as mean (SD). The weighted Kappa test was used to determine the correlation coefficient for agreement between the external evaluators’ lesion whitening scores. A coefficient greater than 0.61 was considered substantial. All analyses were performed with IBM SPSS version 23.

The sample size calculation was performed considering an expected improvement of 50%, 80% power, an alpha error of 0.05, and a confidence interval of 95%.

Using the following Formula 1: 


$$n={\left(Z_{\alpha /2}+Z_{\beta }\right)}^{2}/\Delta xpx\left(1–p\right)$$ (1)

where

*n* is the sample size; Z_α/2_ is the critical value of the normal distribution for a 95% confidence interval (approximately 1.96); Z_β_ is the critical value for 80% power of the test (approximately 0.84); Δ is the minimum detectable, (0.50 in this case); and *p* is the expected proportion (50% or 0.50), a sample size of 32 ~was deemed necessary.

## RESULTS

A total of 33 “before and after” photographic records from vascular lesions in the face were analyzed in this study.

**Figure 1:**
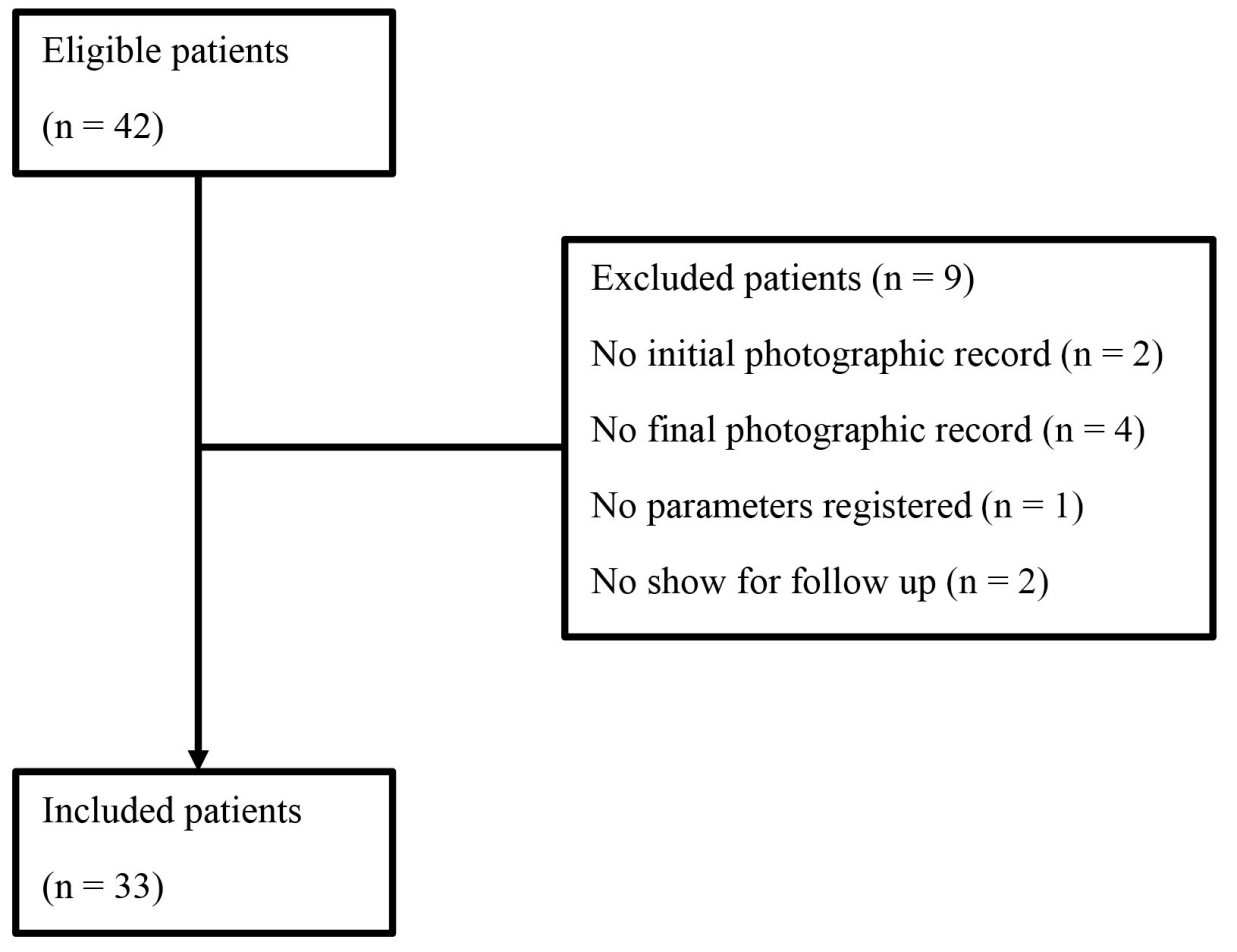
Flow chart illustrating participants included and excluded and final sample size.

The flowchart (Figure 1) shows the total sample, included and excluded cases, and our losses during the follow-up period. Forty-two patients were included, 7 were excluded due to no show at the follow-up consultation, and 2 were excluded due to inappropriate photo records.

The sample comprised 25 women (79%) and 8 men (21%) with a mean age of 42.3 years (range, 19-74 years). The Fitzpatrick classification was type I for 91% of the sample (30/33 subjects).

Perinasal telangiectasia was the most common lesion treated, accounting for 54% of the sample (18/33 lesions) and 88% of all lesions were treated with a single session (29/33). Table 1 shows the lesion types, location on the face, and other epidemiological data.

**Table 1:** Characteristics of the study population.

**Variable**	**N=34**
Age	42.3 ± 13.9
Female, n (%)	25 (73.5)
Fitzpatrick classification, n (%)	
type I	30 (88.2)
type II	2 (5.9)
type III	2 (5.9)
Lesion type, n (%)	
Telangiectasia	27 (79.4)
Reticular veins	4 (11.8)
Venous malformations	3 (8.8)
Lesion site, n (%)	
Nose	18 (52.9)
Infraorbital	6 (17.6)
Temporal area	2 (5.9)
Perioral	2 (5.9)
Chin	2 (5.9)
Lateral cheeks	2 (5.9)
Lips	2 (5.9)

A small spot size (2 mm) was employed for all telangiectasias cases – 76% (25/33 lesions), and a large spot size (6mm) was used for reticular and venous malformations – 24% (8/33 lesions). Pulse duration ranged from 10 to 20 milliseconds for telangiectasias and from 30 to 50 milliseconds for reticular veins and venous malformations. Fluency (joules/cm) ranged from 150 to 175 for telangiectasias and from 50 to 70 for reticular veins and venous malformations.

Despite the risk of complications, there were no adverse effects regarding treatment: no skin burns, no pigmentation, and no mucosa ulceration after the procedures.

According to the photographic records, all lesions had whitened after treatment. The two evaluators scored lesion whitening as 3 or greater on the 0-5 scale in 70 and 88% of patients respectively.

The great majority of patients were treated in a single session (29/33 - 85%); 65% were fully satisfied with the treatment and 35% were partially satisfied, as detailed in Table 2, according to 3 possible alternatives: fully satisfied, partially satisfied, and unsatisfied.

**Table 2:** Number of sessions and patient satisfaction with treatment.

**Number of sessions, n (%)**	
Single session	29 (85.3)
Two sessions	5 (14.7)
Patient satisfaction, n (%)	
Fully satisfied	22 (64.7)
Partially satisfied	12 (35.3)

There was substantial agreement between the external evaluators, as measured by the Kappa test (0.682 – p<0.001) and shown in Table 3.

**Table 3:** Analysis of strength of agreement between the 2 evaluators (Kappa test).

**Confidence interval 95%**				
	Kappa	Inferior limit	Superior limit	Significance
Linear	0.682	0.539	0.781	P<0.001

Kappa was used because the variable is ordinal. Value of k. Strength of agreement; <0.20. Poor; 0.21-0.40. Fair; 0.41-0.60. Moderate; 0.61-0.80. Good; 0.81-1.00. Very good

**Figure 2:**
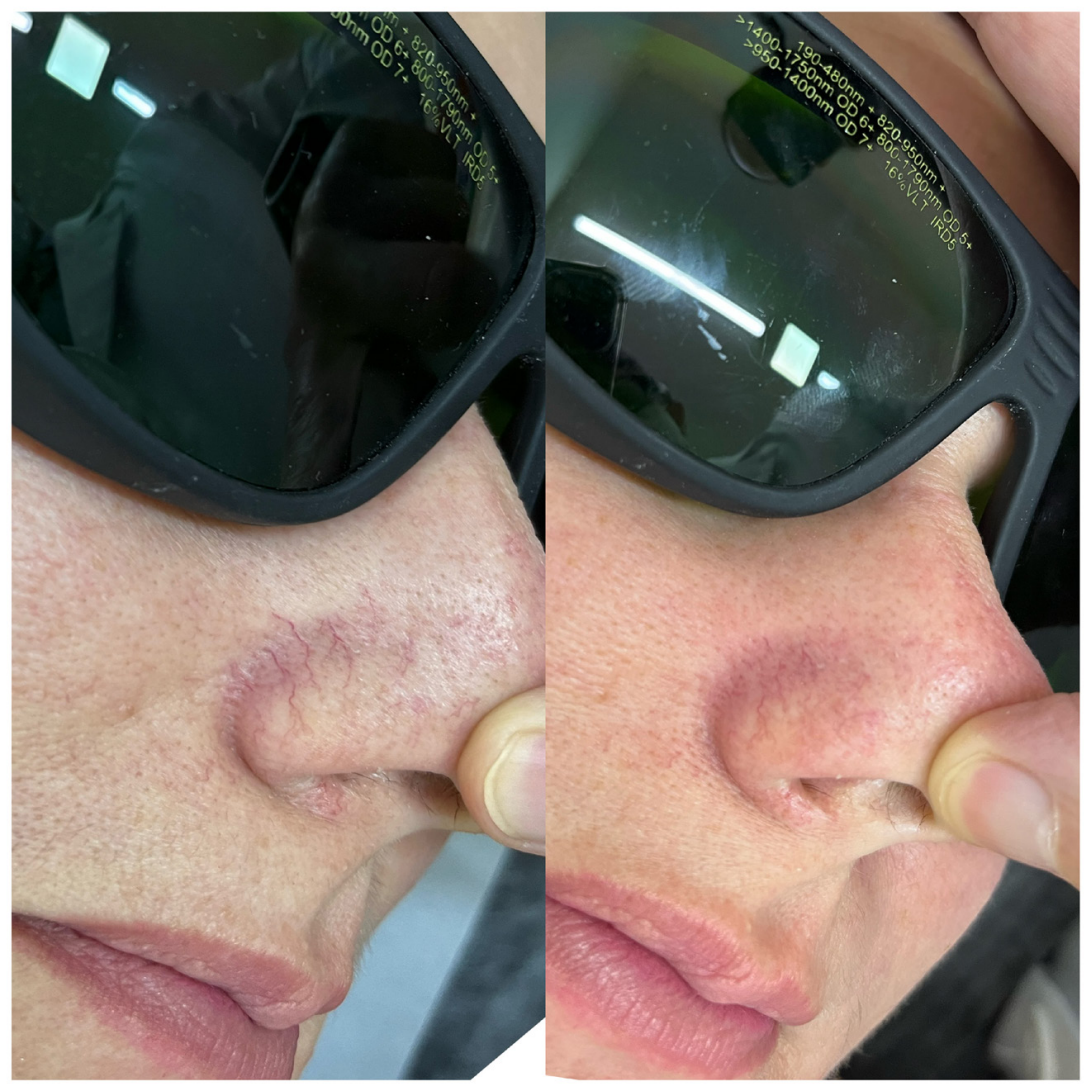
Perinasal telangiectasias treated with Nd YAG 1064nm in a single session. The parameter settings were spotsize 2mm, fluency 150J/cm^2^, and pulse duration 20ms.

**Figure 3:**
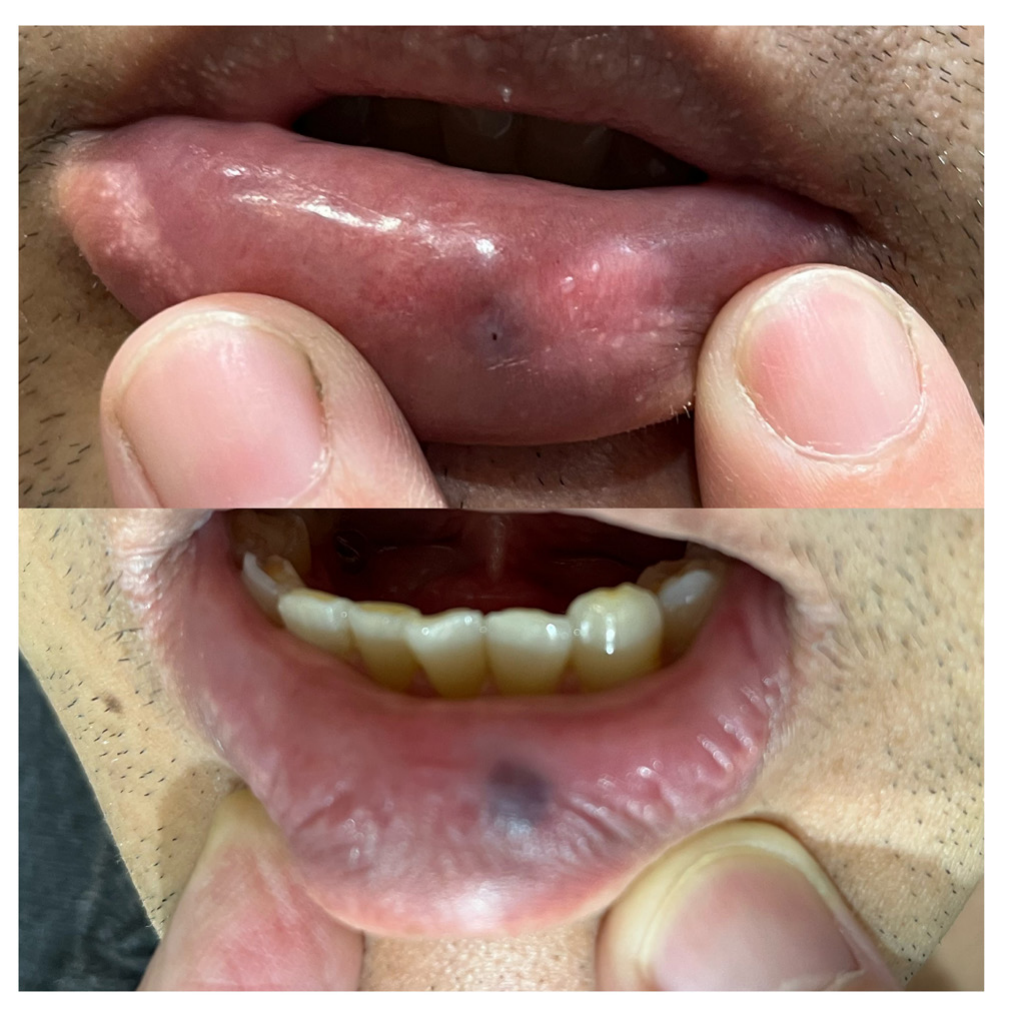
Labial venous malformation treatment with Nd YAG 1064nm in a single session. The parameter settings were spotsize 6mm, fluency 50J/cm^2^ and pulse duration 40ms.

Figures 2 and 3 illustrate results of laser therapy for vascular lesions of the face.

## DISCUSSION

Facial vascular lesion treatment has evolved in parallel with developments in lasers devices over the years.

Before the laser era, sclerotherapy proved to be effective and safe, despite potential for sight-threatening complications from periocular vascular manipulation.^6^

Sclerosant injections could reach the central retinal vein, the choroidal vortex veins, or even the cavernous sinus through venous anastomoses.^7^

Laser treatment for telangiectasias presents theoretical advantages compared to sclerotherapy, including telangiectasic matting reaction, hyperpigmentation, and potential allergenic processes.

However, for effective treatment, the laser source should have a specific wavelength for the vessel and should penetrate deeply to reach through its entire diameter. Shorter laser wavelengths heat only the portion of the vessel wall closest to the skin surface, which can result in incomplete thrombosis in larger and deeper vessels.^8^

Laser devices with longer wavelengths, such as Nd:YAG 1064nm, offer a wider range of treatment possibilities, due to their greater ability to penetrate tissues.^9^

Allied to the greater penetration capacity, the spot size, pulse duration, and fluency adjustments of the Nd:YAG 1064nm laser enable treatment of lesions ranging from superficial and small-diameter examples, such as dermal telangiectasias, to reticular varicose veins larger than 2 mm located deeper in the skin.

Another critical aspect of laser treatment is skin cooling. This prevents skin burns and reduces pain associated with the procedure. We described skin cooling pain reduction in a previous publication.^10^

Using a 1064nm Nd:YAG laser to treat vascular lesions, Ozyurt et al. obtained 97% whitening of facial telangiectasias after 3 laser sessions. The parameters they used to treat facial telangiectasias were: 1.5 mm spot size, pulse duration of 40ms, and fluence of 250J/cm^2^.^11^

Clark et al.^12^ reported on use of the KTP Nd:YAG laser for treatment of superficial cutaneous vascular lesions, describing marked improvement or clearance in 84% of lesions.

Facial telangiectasia treatments have been described by others. Lesion whitening after laser therapy was reported as good to excellent improvement.^13-15^

The present study is in conformity with previous publications, reaching a high grade of whitening for all types of vascular lesions in the face. According to our experience, laser treatment of telangiectasias, reticular veins, and venous malformations is safe and offers excellent results.

Most important is choosing the appropriate laser parameters. Laser devices with shorter laser wavelength can treat superficial lesions but should be avoided for treating reticular veins. Small-size veins have short thermal relaxion times and low energy absorption capacity, demanding short pulse duration, and higher fluences to respond to treatment.

On the other hand, large veins such as infraorbital veins and temporal veins have large thermal relaxion times and high energy absorption capacity, demanding large pulse duration and lower fluences when treated.

Labial venous malformations are dark in color and large in diameter, which characteristics are favorable for laser treatment. Lower fluence with large pulse duration, associated with large spot sizes, offer excellent results with one or two angulated shots per session.

A metanalysis of lower limb telangiectasia treatment analyzed different sclerosant solutions, laser therapy, and foam and concluded that more studies are required to evaluate treatments and outcomes, particularly recurrence, time to resolution, and delayed adverse events.^16^

A systematic review and metanalysis of facial erythema treatment with intense pulsed light (IPL) or pulsed dye laser (PDL) found that IPL significantly improved facial erythema compared to no treatment, with no significant difference between IPL and PDL treatment. The authors concluded that IPL could effectively and safely improve facial erythema with similar efficacy to PDL.^17^

Vascular lesions in the face are common and frequently cause cosmetic issues. Phlebologists and Vascular Surgeons dedicated to treating venous diseases may meet this complaint among their office patients and must be able to offer a safe and effective treatment.

This study was not designed to evaluate safety or efficacy and although our sample did achieve the calculated sample size, its size was small, and this is a limitation to drawing unequivocal conclusions.

Notwithstanding, our experience with this treatment modality and the data from our study show excellent results in terms of whitening, no major complications such as pigmentation, skin burns or matting, treatment required a small number of sessions, and patients’ satisfaction was high, in accordance with previously published studies.

Further studies will contribute to reinforcing the role of transdermal laser in treatment of facial vascular lesions.

## Data Availability

Available upon request: “The data supporting this study are available upon request to the corresponding author, FCN, due to privacy”
